# Accelerated first-pass perfusion CMR using compressed sensing with regional spatiotemporal sparsity

**DOI:** 10.1186/1532-429X-15-S1-E16

**Published:** 2013-01-30

**Authors:** Xiao Chen, Michael Salerno, Frederick H Epstein

**Affiliations:** 1 Biomedical Engineering, University of Virginia, Charlottesville, VA, USA; 2 Medicine, University of Virginia, Charlottesville, VA, USA; 3 Radiology, University of Virginia, Charlottesville, VA, USA

## Background

CMR perfusion images demonstrate complex dynamic behavior resulting from signal intensity changes during the first pass of gadolinium as well as motion from imperfect breathholding and gating. Reconstruction algorithms such as *kt*-PCA and compressed sensing (CS) techniques such as *kt*-Sparsity and Low-Rankness (*kt*-SLR) assume that a few spatiotemporal basis functions can model this intricate behavior [1,2]. However, these techniques are sensitive to motion, as the basis functions do not accurately describe the complete dynamics of the entire image set. We propose a novel method that utilizes regional sparsity by dividing the images into regions. With this approach, the simplified dynamics of smaller regions can be better described by a limited number of basis functions. This method was tested on *in vivo* images and simulated data, and the results were compared to *kt*-SLR [1], a CS method that uses global sparsity.

## Methods

Images were spatially divided into square blocks (approximately 15×15 pixels). As small blocks have simpler dynamic patterns and are insensitive to dynamic changes in other regions of the image, they can be represented with fewer basis functions. Singular value decomposition was applied to the dynamic blocks to exploit the high spatiotemporal correlations within them. Iterative soft thresholding [3] was applied to filter low singular values, which primarily represent incoherent noise and aliasing. The de-aliased blocks were merged back into images using weighted averaging [4]. Images underwent iterative CS reconstruction through the blocking, thresholding and merging procedures, subject to fidelity with collected k-space data. Four first-pass datasets (chosen to have prominent respiratory motion) and a simulated phantom featuring respiratory motion and time-varying signal intensity were retrospectively undersampled at an acceleration rate of 4 and reconstructed using the regional sparsity method and *kt*-SLR. Mean square error (MSE) was calculated for quantitative analysis.

## Results

**Figure 1:**
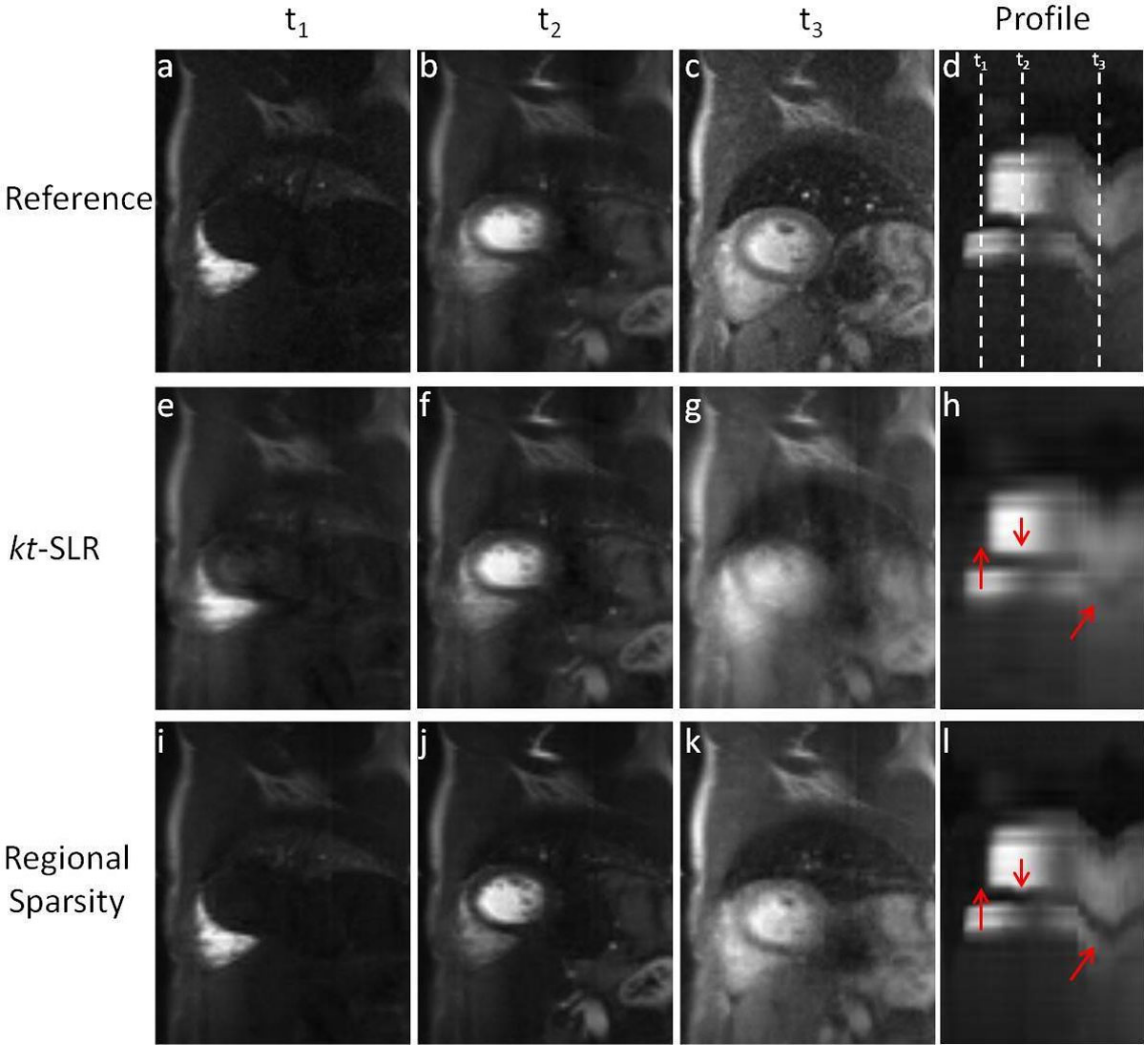
Example images at different time points and temporal profiles from *in vivo* patient perfusion CMR. Fully sampled data (a-d) serve as a reference and time points are pointed out on profiles(d). The proposed regional sparsity method (i-l) outperformed *kt*-SLR (e-h) at rate 4 acceleration. More residual artifacts were found on *kt*-SLR, where the difference was most obvious at t_3_ when large motion occurred (k v.s. g). The temporal profiles also show that using regional sparsity better recovered the motion than *kt*-SLR, as highlighted by red arrows.

Figure 1 shows example results from *in vivo* imaging. Example images and spatiotemporal profiles at certain time points where motion occurred show that the proposed method is substantially less sensitive to motion than *kt*-SLR. At these time points, less blurring and streaking artifacts were observed when employing regional sparsity. Average MSE for *in vivo* images using regional sparsity and *kt*-SLR were (10.0±2.3)×10^-7^ and (20.7±5.4)×10^-7^, respectively. Images from the simulated phantom showed MSE was (3.7±0.6)×10^-7^ and (6.7±1.8)×10^-7^ for regional sparsity and *kt*-SLR, respectively.

## Conclusions

A novel CS method using regional sparsity was less sensitive to motion than *kt*-SLR for CMR perfusion imaging. Future work includes developing improved regional separation methods, such as pattern recognition, and further improved motion compensation using regional motion tracking.

## Funding

This study is funded by Siemens Medical Solutions, NIH R01 EB 001763 and American Heart Association Predoctoral Award 12PRE1204005
